# Reactive Infectious Mucocutaneous Eruption (RIME) in a Teenage Male: Diagnostic Challenges in a Resource-Limited Setting

**DOI:** 10.7759/cureus.110187

**Published:** 2026-06-03

**Authors:** Moshanti M Ramdath, Joel D Teelucksingh, Samuel Aboh, Meghan Lalla, Renisha Bisnath, Nicholas R Stephen, Celina Ramjattan

**Affiliations:** 1 Internal Medicine/Endocrinology, San Fernando General/Teaching Hospital, San Fernando, TTO; 2 Internal Medicine, San Fernando General/Teaching Hospital, San Fernando, TTO; 3 Infectious Diseases, San Fernando General/Teaching Hospital, San Fernando, TTO

**Keywords:** mucositis, mycoplasma, mycoplasma pneumoniae–induced rash and mucositis (mirm), pneumonia, reactive infectious mucocutaneous eruption (rime)

## Abstract

*Mycoplasma pneumoniae* is a common cause of community-acquired pneumonia with both pulmonary and extrapulmonary manifestations. Reactive infectious mucocutaneous eruption (RIME) is a recently distinguished, distinct mucocutaneous syndrome characterized by prominent mucositis with or without cutaneous involvement. Differentiating RIME from other severe mucocutaneous conditions, such as Stevens-Johnson syndrome (SJS) and toxic epidermal necrolysis (TEN), is critical due to differences in prognosis and management. We report the case of a previously healthy teenage male presenting with a biphasic illness characterized by respiratory symptoms followed by severe mucositis and limited, delayed cutaneous involvement. In a resource-limited setting where confirmatory tests were delayed, diagnosis relied heavily on clinical recognition and multidisciplinary input. The patient was successfully managed with antimicrobial therapy, systemic corticosteroids, and supportive care, resulting in full recovery. This case highlights the critical importance of multidisciplinary care, particularly in complex mucocutaneous syndromes where diagnostic uncertainty exists. RIME should be considered in adolescents presenting with a respiratory prodrome followed by pronounced mucositis and minimal cutaneous involvement. Increased clinician awareness and further prospective studies are needed to establish validated diagnostic criteria and evidence-based treatment guidelines.

## Introduction

Reactive infectious mucocutaneous eruption (RIME) is a recently coined umbrella term, following the evolution of *Mycoplasma*-induced rash and mucositis (MIRM) over the past decade [1]. It became evident that clinical presentations consistent with MIRM can occur in response to other infective triggers [2]. 

RIME commonly affects mucous membranes with minimal or no cutaneous involvement(<10% body surface area, BSA) [3]. It occurs predominantly in males (66-74%), with a mean age of 11.9-16 years, primarily affecting children and adolescents [2,4,5]. RIME has also been reported to occur in adults [2].

RIME must be differentiated from other conditions with similar mucocutaneous involvement, such as urticaria, erythema multiforme, Stevens-Johnson syndrome (SJS), drug reaction with eosinophilia and systemic symptoms (DRESS), and toxic epidermal necrolysis (TEN) [3]. It presents clinically with a prodromal phase lasting approximately seven to nine days and defined by upper respiratory tract symptoms or conjunctivitis, followed by extensive mucosal involvement [3]. However, the prodromal phase can also be absent [3].

Mucosal involvement in RIME is characterized by oral (94-96.3%), urogenital (59.3-63%), and ocular (82-92.6%) involvement and, less commonly, anal or esophageal surfaces [1]. Manifestations include accompanying ulcerations, erosions with denudation, hyperemia, and pseudomembrane formation [1].

Despite increasing recognition, RIME remains underdiagnosed and frequently misclassified due to its similar presentation to SJS/TEN, DRESS, and other dermatoses, particularly in resource-limited settings where confirmatory testing is delayed or unavailable.

## Case presentation

A previously healthy 16-year-old male presented to the emergency department (ED) with a 10-day history of fever, productive cough (yellow sputum), bilateral conjunctival injection, and a six-day history of painful oral ulcers. He also experienced joint pains, generalized headaches, decreased appetite, body pains, dysphagia, and odynophagia to both solids and liquids.

He had previously visited a general physician and was prescribed a five-day course of amoxicillin-clavulanic acid, of which he completed two days of treatment. He then presented to the ED due to worsening symptoms. There was no rash, neck stiffness, diarrhea, palpitations, dysuria, hematuria, nausea, or vomiting.

There was no history of sick contacts, recent travel abroad, allergies, recent vaccinations, herbal or over-the-counter medications, exposure to new medications (except those prescribed by his general practitioner), or illicit drug use. He was not sexually active. There was no family history of inflammatory bowel disease or autoimmune conditions.

**Figure 1:**
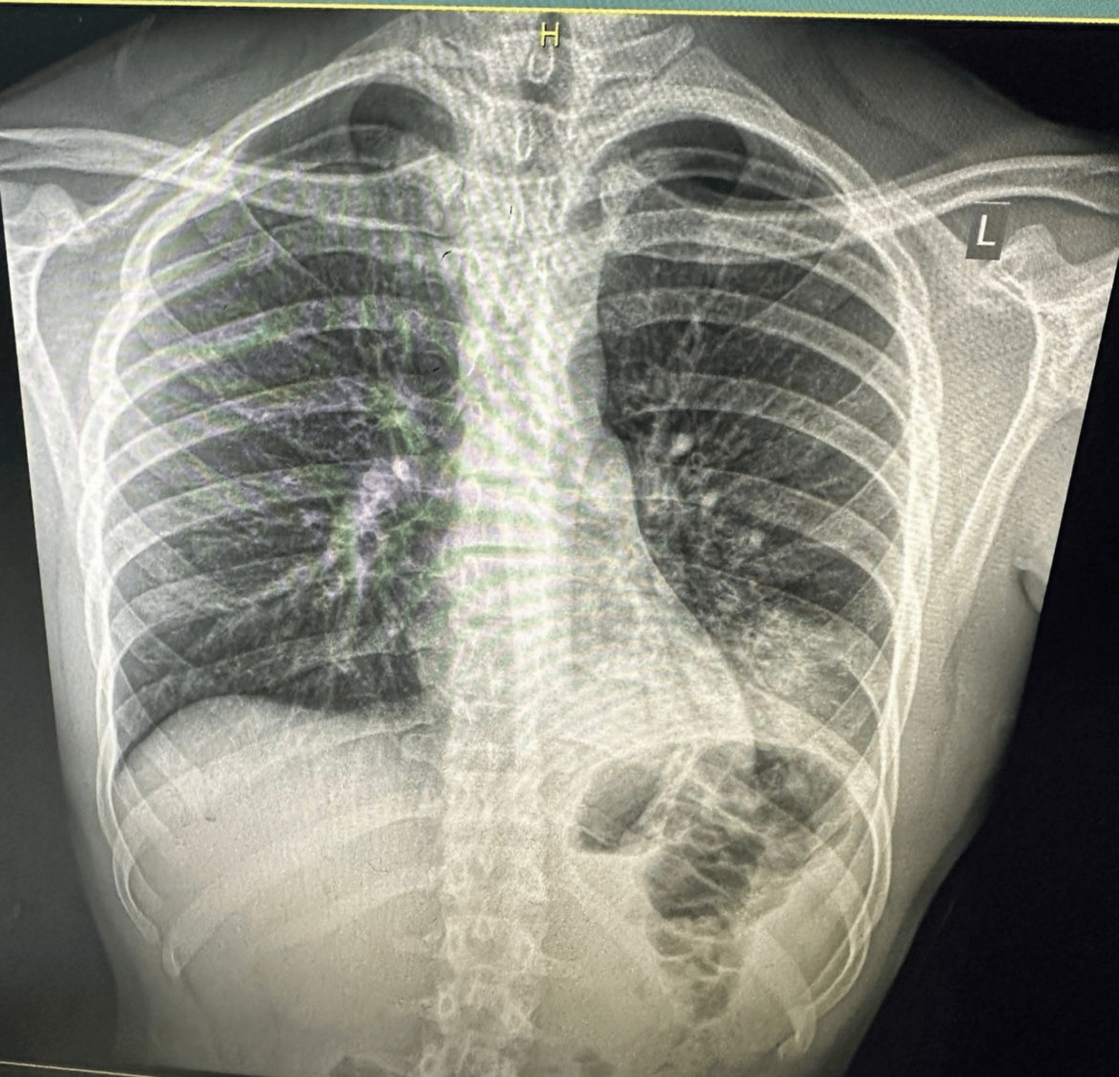
Anteroposterior view of chest X-ray on admission: bilateral, predominantly lower zone patchy airspace/interstitial infiltrates, left > right, with a patchy consolidation in the left lung base.

On examination, he appeared dehydrated with a blood pressure of 114/54 mmHg, a pulse of 64 bpm, oxygen saturation of 95% on room air, and a temperature of 38℃. On physical examination, he had oral ulcers, bilateral conjunctivitis (no discharge), no rashes or genital ulceration, and no lymphadenopathy. Nikolsky's sign was negative. Left basal crepitations were elicited on the respiratory examination; cardiovascular, abdominal, and neurological examinations were unremarkable. Laboratory findings demonstrated leukocytosis with neutrophilia and elevated inflammatory markers (Table 1). Chest radiography revealed bilateral lower-zone infiltrates consistent with an atypical pneumonia (Figure 1).

**Table 1:** Hematological and biochemical investigations on admission. ANA: antinuclear antibody, ANF: antinuclear factor, VDRL: Venereal Disease Research Laboratory

Test	Value	Reference range
White blood cell count	17.01 × 10³/µL	4.1-11.2 × 10³/µL
Neutrophils	88.9%	39.9-73.9%
Lymphocytes	4.5%	18.8-50.8%
Hemoglobin	13.2 g/dL	11.7-15.5 g/dL
Platelet count	237 × 10³/µL	159-388 × 10³/µL
Sodium	137 mmol/L	135-145 mmol/L
Potassium	3.9 mmol/L	3.5-5.1 mmol/L
Chloride	96 mmol/L	97-110 mmol/L
Blood urea nitrogen	12 mg/dL	6-24 mg/dL
Creatinine	0.6 mg/dL	0.7-1.2 mg/dL
C-reactive protein	35.951 mg/dL	0.1-0.5 mg/dL
Erythrocyte sedimentation rate	48 mm/hr	15-20 mm/hr
Aspartate aminotransferase	23.9 IU/L	5-40 IU/L
Alanine aminotransferase	25.9 IU/L	5-41 IU/L
Gamma-glutamyl transferase	22 U/L	6-61 U/L
ANA/ANF	Negative	Negative at 1:100 dilution
Respiratory panel SARS-CoV-2	Not detected	Not detected
VDRL (serum)	Non-reactive	Non-reactive

At presentation, differential diagnoses included SJS, erythema multiforme, viral mucositis, Kawasaki disease, and autoimmune conditions, such as systemic lupus erythematosus. However, the biphasic presentation and predominant mucosal involvement raised early suspicion for RIME. The oral ulcers were also present prior to initiating the outpatient amoxicillin-clavulanic acid, which further supported the diagnosis of RIME (Table 2). 

**Table 2:** Timeline of presentation of signs and symptoms.

Day of illness	Event
Day 1	Fever + cough
Day 4	Oral ulcers and conjunctivitis
Day 10	Hospital presentation
Day 14	Rash appears
Day 23	Patient discharged home

Infectious disease (ID) review suggested a diagnosis of likely RIME secondary to *M. pneumoniae*. He was managed with azithromycin 500 mg (orally), ceftriaxone 1 g (IV), and systemic corticosteroids (hydrocortisone 100 mg IV every eight hours), in addition to fluids, analgesia, and nutritional support. Sputum for MCS (microscopy, culture, and sensitivity), respiratory panel, viral screen, and *Mycoplasma* PCR were requested, the latter of which had to be facilitated at a private laboratory (Tables 1, 3).

**Table 3:** Serological investigations.

Test	Value (U/mL)	Negative	Borderline	Positive
Coxsackie and Echovirus IgG	0.42	<0.8	0.8-1.1	>1.1
Coxsackie and Echovirus IgM	0.84	<0.8	0.8-1.1	>1.1
Epstein–Barr virus IgG	7.53	<16	16-22	>22
Epstein–Barr virus IgM	0.56	<0.8	0.8-1.1	>1.1
Mycoplasma pneumoniae IgM	4.96 (High)	<0.8	0.8-1.1	>1.1
Herpes simplex virus-1 IgG	4.5	<20	20-25	>25
Herpes simplex virus-1 IgM	21.8	<20	20-25	>25
Herpes simplex virus-2 IgG	5.5	<20	20-25	>25
Herpes simplex virus-2 IgM	15.2	<20	20-25	>25

**Figure 2:**
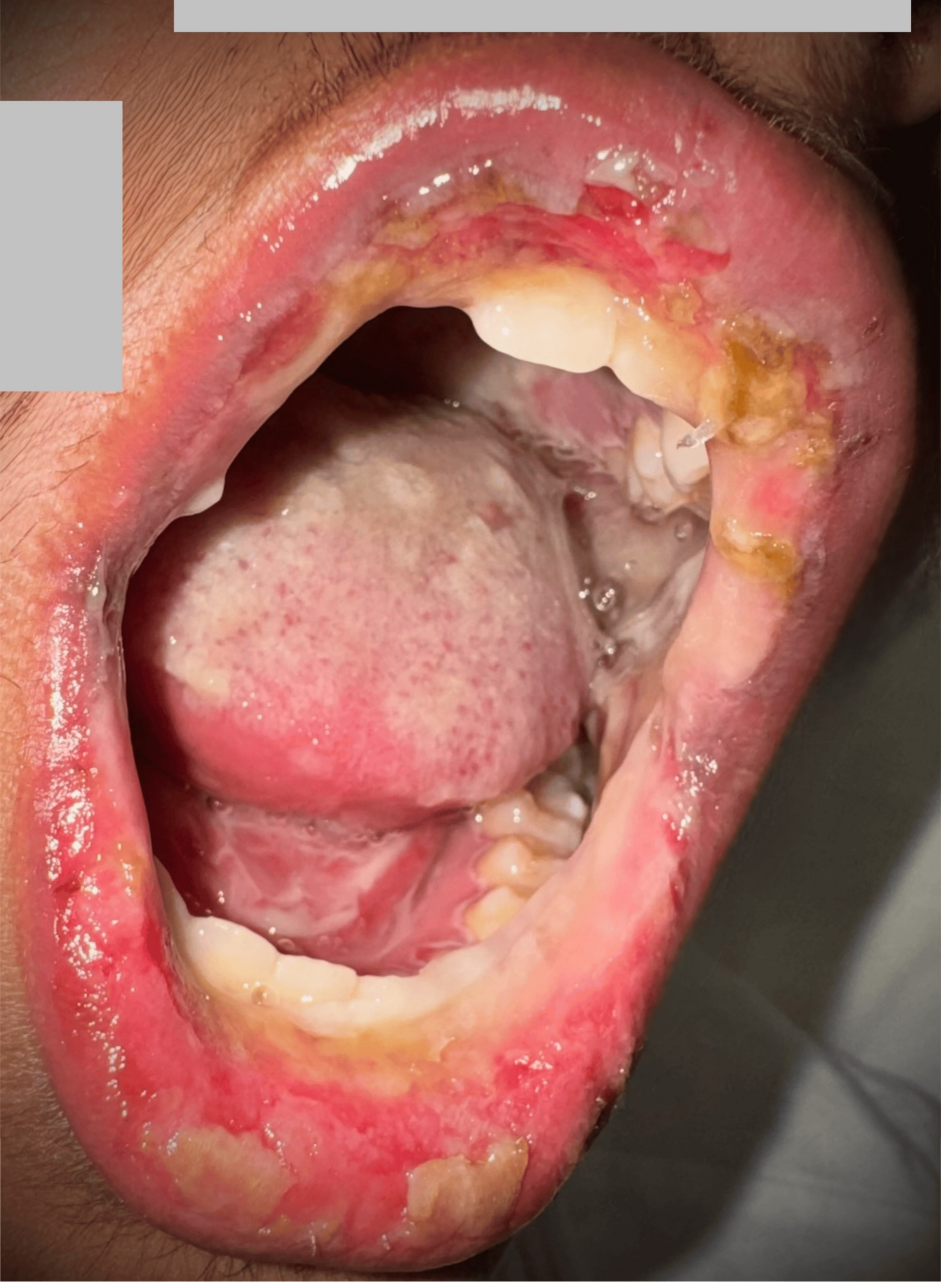
Extensive erosions and shallow ulcerations were seen involving the labial and oral mucosa, with whitish-yellow exudate and posterior oropharyngeal erythema. The tongue was diffusely coated with underlying erythema and prominent papillae, consistent with severe erosive mucositis.

**Figure 3:**
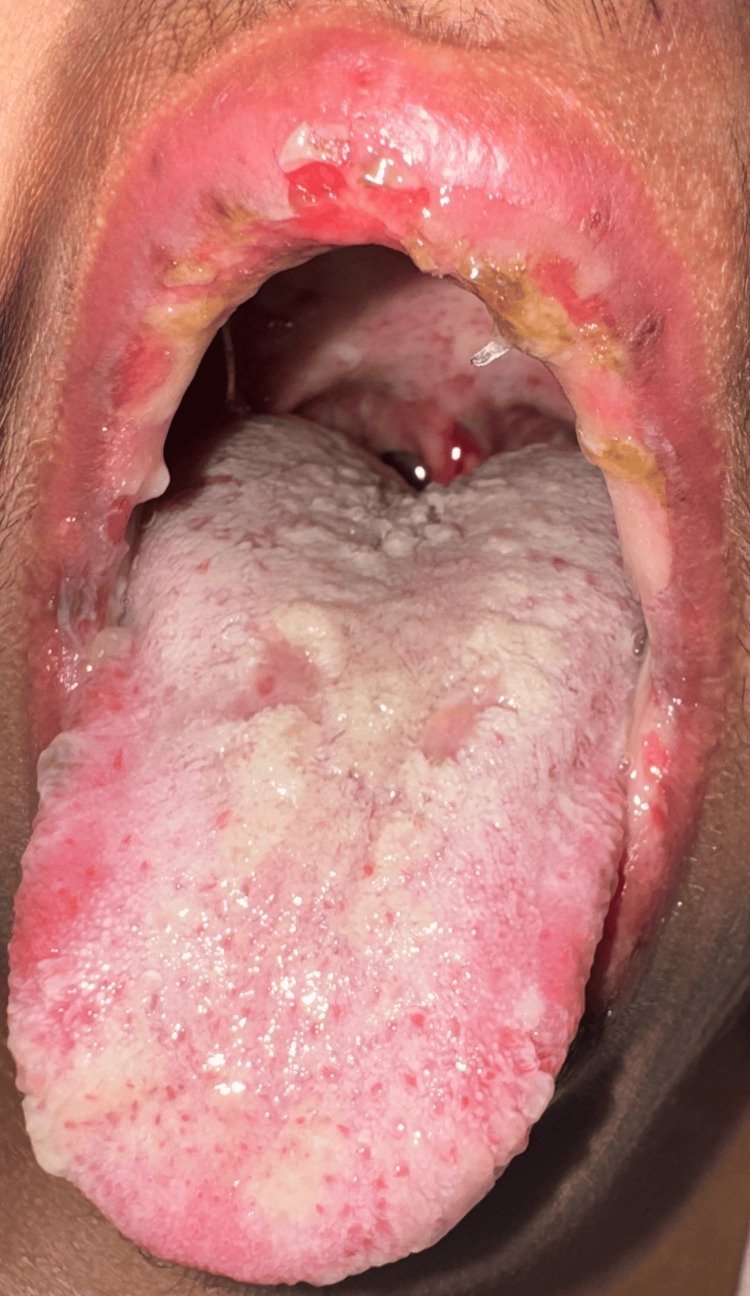
The lips were erythematous, swollen, and crusted, with yellowish slough and hemorrhagic crusting along the vermilion border.

Otorhinolaryngology (ORL) review confirmed severe oral mucositis with white patches covering the tongue, uvula, pharynx, and the hard and soft palate with crusting, erythematous tonsils, and ulceration of the pharynx, oral, and buccal mucosa (Figures 2, 3). He was placed on a mix containing lidocaine, diphenhydramine, and prednisolone (magic mouthwash solution).

**Figure 4:**
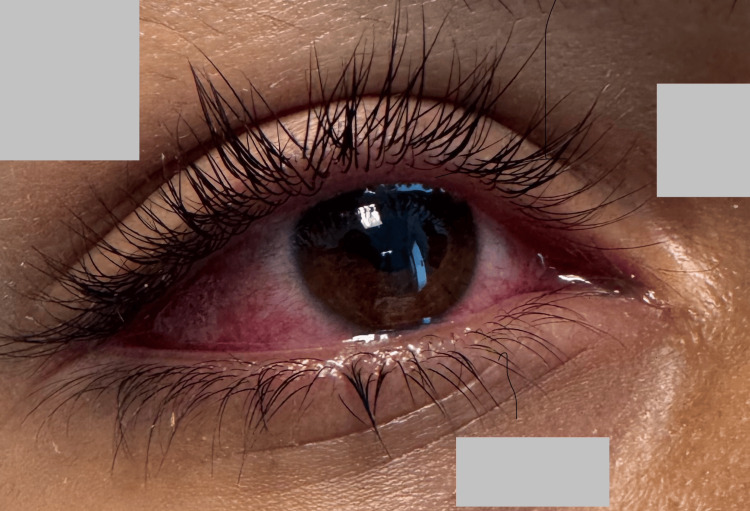
Right eye with conjunctival injection, associated mild eyelid erythema and no purulent discharge.

Ophthalmology review confirmed the presence of bilateral conjunctivitis (Figure 4); sodium hyaluronate eye drops 4% and prednisolone acetate 1% eye drops were prescribed.

**Figure 5:**
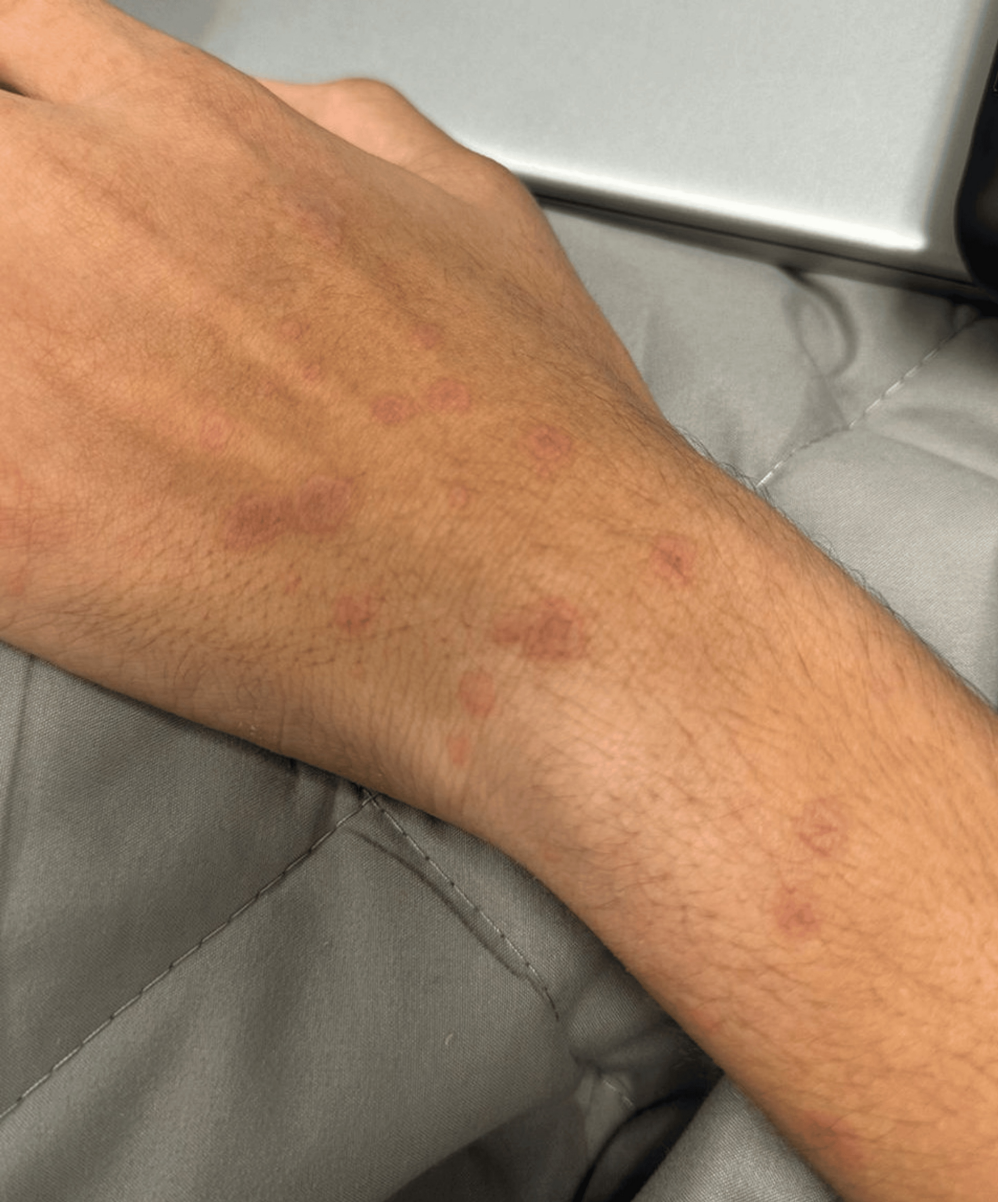
Atypical targetoid macules and papules on the dorsum of the left hand.

Dermatology review also confirmed the presence of mucositis. On day 4 post admission, sparse, discrete targetoid lesions appeared in the form of erythematous, blanching, non-pruritic, non-tender macules and papules on the extremities with centripetal extension to the trunk and arms (Figure 5).

At this stage, the patient was tolerating small amounts orally with gradual resolution of symptoms, such as the productive cough, conjunctivitis, and fever. A three-day course of azithromycin and a five-day course of ceftriaxone were completed. ID team recommended that ceftriaxone be continued and a five-day course of levofloxacin (500 mg) be prescribed upon discharge with outpatient follow-up. ORL, Ophthalmology, and Dermatology continued previous management with the addition of antihistamines.

**Figure 6:**
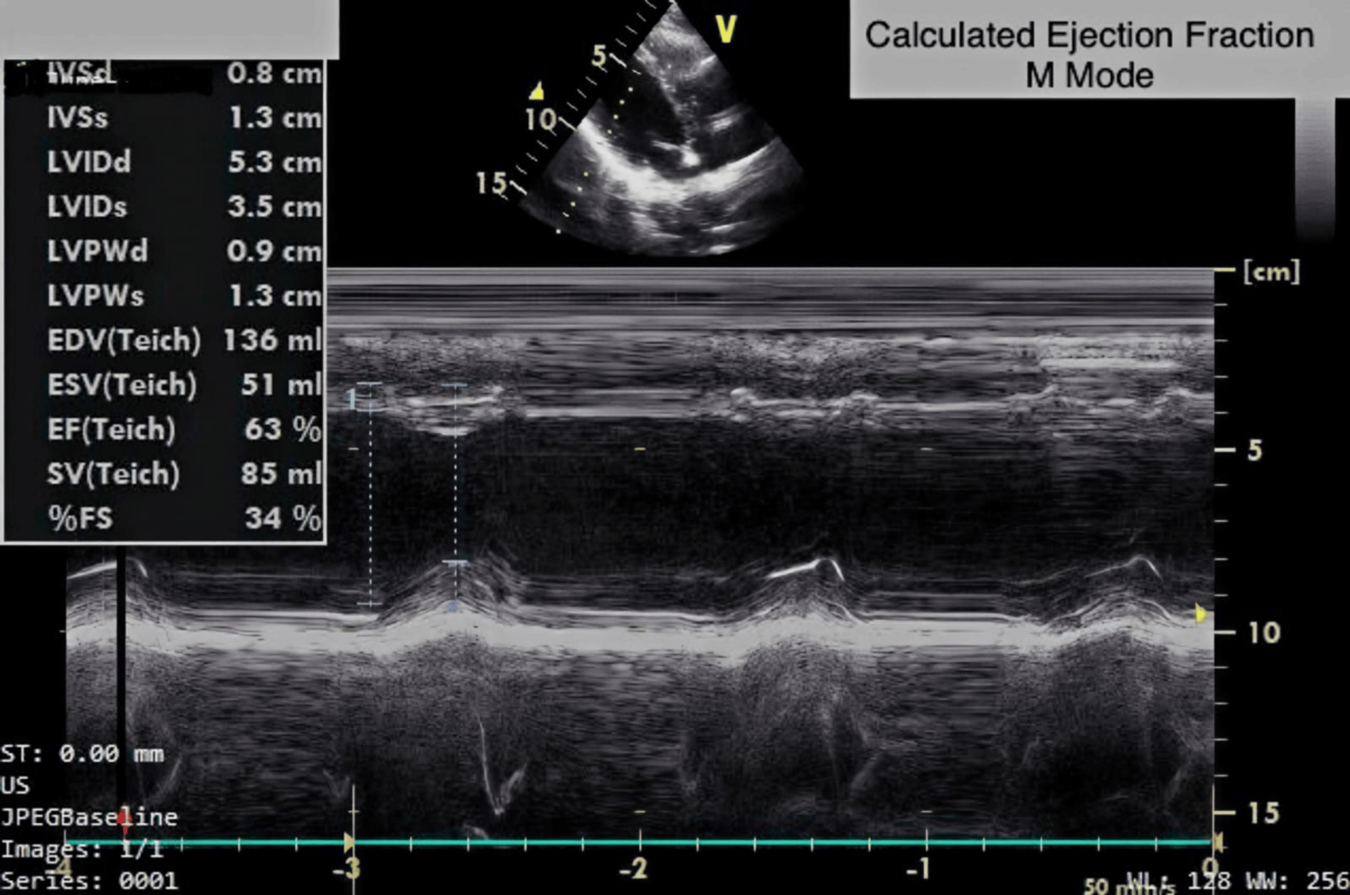
Calculated ejection fraction: M mode.

**Figure 7:**
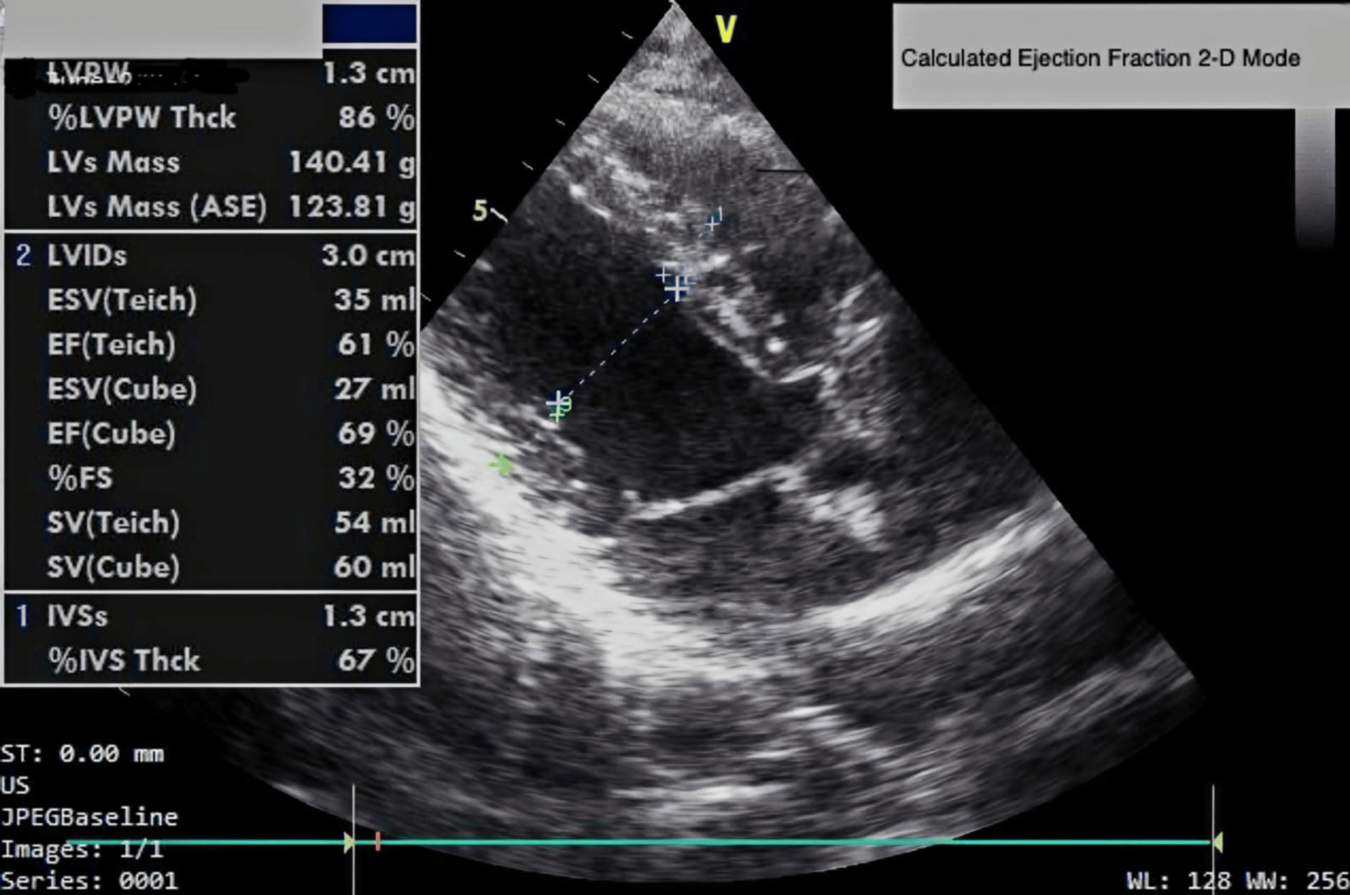
Calculated ejection fraction: 2D mode.

**Figure 8:**
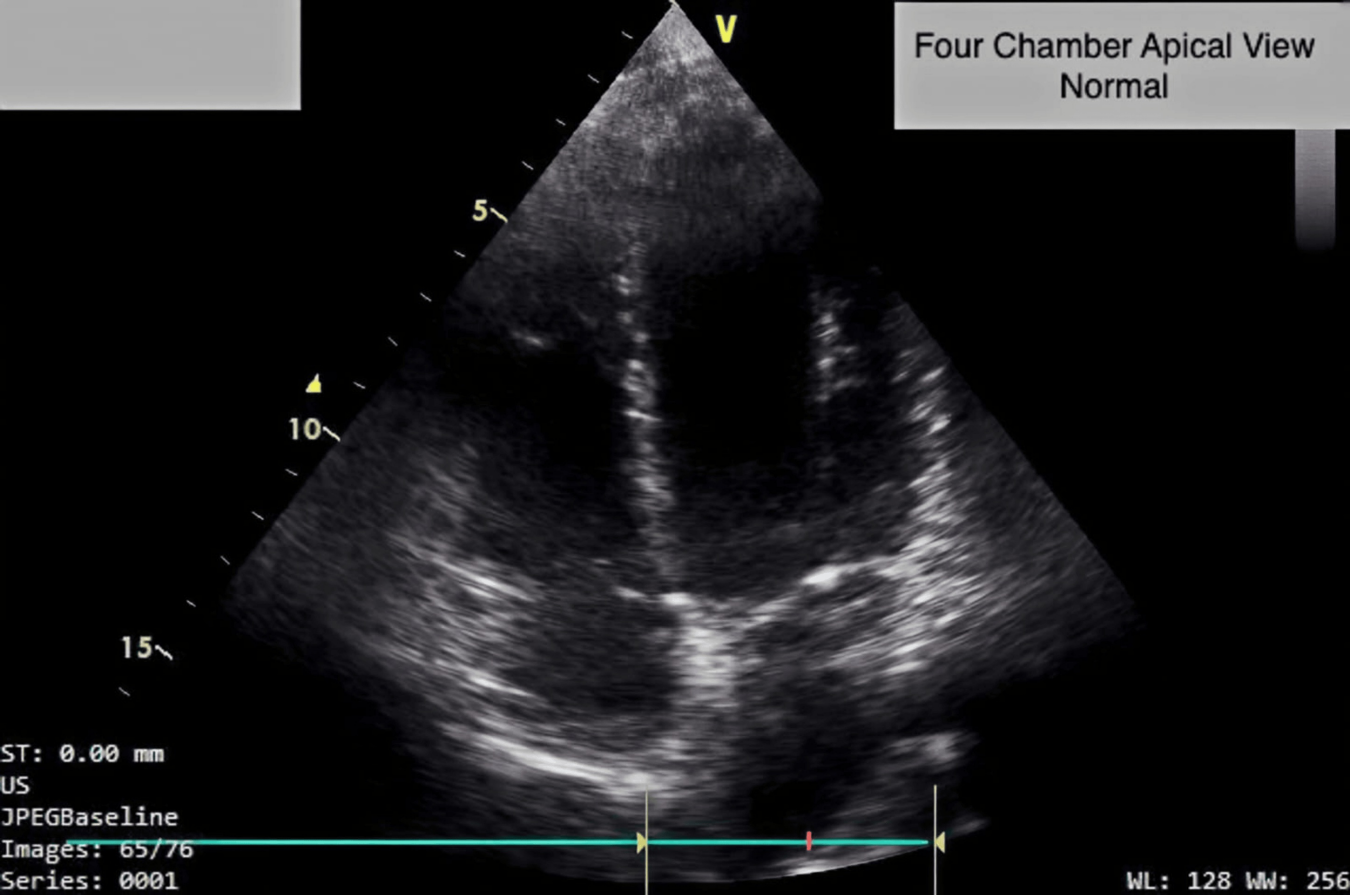
Four-chamber apical view: normal.

Laboratory investigations revealed the following: alpha hemolytic streptococci on sputum culture (normal flora), rapid HIV was negative, and blood and urine cultures yielded no bacterial growth. On day 10 post admission, a respiratory panel only yielded a positive result for *M. pneumoniae *via PCR. SARS-CoV-2 was negative. Serology for EBV, CMV, HSV, and syphilis was negative; ANA/ANF titres were also negative (Table 2). The echocardiogram was structurally normal (Figures 6-8).

This patient improved with the combined therapy. The conjunctivitis resolved, the rash gradually subsided, and the mucositis significantly improved, allowing resolution of dysphagia. He was subsequently discharged on day 13 post-admission in a stable condition to be followed up in ORL, Ophthalmology, ID, and Dermatology outpatient clinics (over three months). Oral prednisolone (30 mg) was tapered over three weeks.

## Discussion

MIRM was initially identified as a distinct clinical entity by Canavan et al. in 2015 [4]. Gradually, it became evident that clinical presentations consistent with MIRM can occur in response to other infective triggers [2]. When MIRM was initially coined, *M. pneumoniae *was identified as the sole trigger. However, although it remains the most commonly reported trigger, other infectious etiologies have since been described, including *Chlamydia pneumoniae*, human metapneumovirus, human parainfluenza virus 2, rhinovirus, enterovirus, influenza A and B, coronavirus disease 2019 (COVID‐19), and adenovirus [3]. This led to the proposal of this distinct unifying term called RIME by collaboration within the Pediatric Dermatology Research Alliance in 2020 [6].

This evolving nomenclature reflects the increasing recognition of similar clinical phenotypes associated with other pathogens, while maintaining the clinical relevance of RIME as a distinct and well-described entity [3]. The proposed RIME criteria include evidence of an infectious trigger in addition to two of the following: insignificant medication history, erosive mucositis involving two or more sites, and lesions that are vesiculobullous or atypical targets with less than 10% BSA involvement [7]. Additional supportive features include the presence of prodromal symptoms and histological findings [7]. Our patient met these criteria with confirmed *M. pneumoniae *via PCR, absence of significant medication history, erosive mucositis at ≥2 sites (oral and ocular), and the presence of atypical targetoid lesions with <10% BSA involvement. The presence of prodromal symptoms further supported the diagnosis of RIME. 

While the exact mechanism of RIME remains unclear, these immune-mediated pathways likely explain the prominent mucosal involvement. It is proposed that immune activation, antibody production, immune complex deposition, and complement activation can result in mucocutaneous manifestations [8]. Other proposed mechanisms include molecular mimicry between *Mycoplasma* P1-adhesion molecules and host keratinocytes with resultant injury via cytotoxic T cells or antibodies [8]. By contrast, the pathogenesis of SJS/TEN is thought to involve a type IV hypersensitivity reaction mediated by Fas-Fas ligand keratinocyte apoptosis [9].

Distinguishing RIME from other mucocutaneous eruptions is crucial but sometimes challenging. RIME is associated with prodromal symptoms such as malaise, fever, and cough roughly one week before the appearance of the rash [3]. SJS/TEN is also associated with prodromal symptoms and upper respiratory tract infection [10]. However, there is usually a history of recent drug exposure, such as antibiotics, HAART (highly active antiretroviral therapy), NSAIDs (non-steroidal anti-inflammatory drugs), and antiepileptics [10]. Our patient had no significant drug exposure prior to presentation. 

Mucosal involvement is the hallmark of RIME, with the greatest involvement occurring in the oral mucosa, followed by the ocular region, then the urogenital region [1]. As seen in our patient, there was significant mucositis involving the oral and buccal mucosa. There was also bilateral conjunctivitis without ulceration or purulent discharge. The early predominance of mucositis preceding cutaneous involvement, in the absence of significant drug exposure, favored RIME over SJS.

In addition, cutaneous morphology is important in distinguishing RIME from SJS/TEN. The cutaneous manifestations in SJS/TEN are characterized by macules, atypical target lesions, purpura, erythema, and numerous flaccid blisters [10]. In addition, in SJS, there is <10% skin detachment, whereas skin detachment involving 10-30% BSA overlaps between SJS and TEN, and > 30% BSA meets the criteria for TEN [10]. A positive Nikolsky sign is also highly suggestive of SJS/TEN [11]. The Nikolsky sign was negative in our patient.

In our patient, the herpes simplex virus-1 (HSV-1) IgM titres and Coxsackie and Echovirus IgM titres were both borderline. Although both viruses are recognized differentials in patients presenting with mucocutaneous eruptions and epidermal detachment [3], our patient lacked characteristic clinical features suggestive of active infection, such as the vesicular or blistering lesions typically associated with HSV infection, gingivostomatitis, regional lymphadenopathy, or the systemic manifestations commonly seen with Coxsackie or Echovirus infection. As a result, the borderline serological findings were considered more likely to represent non-specific reactivity or serological cross-reactivity rather than active infection. Furthermore, *M. pneumoniae* PCR was positive, providing microbiological confirmation of *M. pneumoniae* infection. 

In the absence of standardized management guidelines, a multidisciplinary approach was adopted to mitigate disease complications. Expert consultations from the ID, ORL, Ophthalmology, and Dermatology specialties played a pivotal role. Management was further guided by symptom severity and diagnostic uncertainty, necessitating a broad supportive and antimicrobial approach. In resource-limited settings such as ours, where viral screening and *Mycoplasma* PCR testing are both costly and subject to significant delays due to the reliance on private labs, clinical judgement played a significant role. Consequently, empiric initiation of treatment for RIME was started early to prevent complications, utilizing the proposed criteria stated above.

Management focused on supportive care, including antibiotic therapy, systemic corticosteroids, adequate analgesia, hydration, and nutritional optimization. Patients experiencing atypical pneumonia symptoms may benefit from treatment with macrolides, tetracyclines, or fluoroquinolones [3]. Our patient received intravenous ceftriaxone and was discharged on a five-day regimen of levofloxacin under close supervision from ID. Although a macrolide would be preferred in a 16-year-old male with *M. pneumoniae *infection and severe mucositis, azithromycin was unavailable (temporarily). Following ID review, levofloxacin was selected as an alternative agent in view of the *M. pneumoniae* susceptibility and concerns regarding medication allergies. Current pediatric guidelines permit use of fluoroquinolones in adolescents when suitable alternatives are unavailable or inappropriate and when the anticipated benefits outweigh potential risks [12]. Our patient was closely monitored and tolerated therapy without documented adverse effects.

Empirical use of corticosteroids, immunosuppressants, and IVIG has been documented in severe cases of RIME [3]; however, their effectiveness remains disputed [13]. Upon admission, our patient was administered intravenous hydrocortisone (100 mg thrice daily) and was discharged on a tapering regimen of prednisolone (commencing at 30 mg over three weeks), which resulted in significant clinical improvement. The prognosis of RIME is favorable, with 81% of patients making a full recovery [1]. Our patient made a full recovery without any complications over the three-month follow-up period. Complications associated with RIME include post-inflammatory hyperpigmentation, lymphopenia, ocular mucosal damage with resultant conjunctival shrinkage, corneal ulceration, and even blindness [1,3]. This case highlights the critical importance of multidisciplinary care, particularly in complex mucocutaneous syndromes where diagnostic uncertainty exists. Early collaboration between specialties facilitated appropriate management and favorable outcomes.

## Conclusions

RIME should be considered in adolescents presenting with mucositis following a respiratory illness, particularly in the absence of significant drug exposure. Prompt multidisciplinary intervention and supportive care can achieve excellent outcomes despite the lack of standardized treatment guidelines. Increased awareness of RIME is critical to avoid misdiagnosis and to facilitate timely, appropriate management, particularly in low-resource environments where clinical recognition remains paramount.

Key limitations of this report include its being a single case report, the absence of a skin biopsy, borderline serological results likely reflecting cross-reactivity, and delayed confirmatory testing.

## References

[1] Damari N, Goodman A, Bridges C (2025). Diagnosis and management of reactive infectious mucocutaneous eruption. J Hosp Med.

[2] Haseeb A, Elhusseiny AM, ElSheikh RH, Tahboub MA, Kwan JT, Saeed HN (2023). Ocular involvement in Mycoplasma induced rash and mucositis: a systematic review of the literature. Ocul Surf.

[3] García-Rodríguez V, Iglesias-Sancho M, Martin-Poch A, Fernández-Figueras MT, Salleras-Redonnet M (2025). Reactive infectious mucocutaneous eruption (RIME): narrative review and proposed management algorithm. Dermatol Pract Concept.

[4] Canavan TN, Mathes EF, Frieden I, Shinkai K (2015). Mycoplasma pneumoniae-induced rash and mucositis as a syndrome distinct from Stevens-Johnson syndrome and erythema multiforme: a systematic review. J Am Acad Dermatol.

[5] Lofgren D, Lenkeit C (2021). Mycoplasma pneumoniae‐Induced rash and mucositis: a systematic review of the literature. Spartan Med Res J.

[6] Ramien ML, Bruckner AL (2020). Mucocutaneous eruptions in acutely ill pediatric patients-think of Mycoplasma pneumoniae (and other infections) first. JAMA Dermatol.

[7] Ramien ML, Bahubeshi A, Lara-Corrales I (2021). Blistering severe cutaneous adverse reactions in children: proposal for paediatric-focused clinical criteria. Br J Dermatol.

[8] Mayor-Ibarguren A, Feito-Rodriguez M, González-Ramos J, Del Rosal-Rabes T, González-Sainz FJ, Sánchez-Orta A, de Lucas-Laguna R (2017). Mucositis secondary to Chlamydia pneumoniae infection: expanding the Mycoplasma pneumoniae-induced rash and mucositis concept. Pediatr Dermatol.

[9] Martínez-Pérez M, Imbernón-Moya A, Lobato-Berezo A, Churruca-Grijelmo M (2016). Mycoplasma pneumoniae-induced mucocutaneous rash: a new syndrome distinct from erythema multiforme? Report of a new case and review of the literature. Actas Dermosifiliogr.

[10] Chatproedprai S, Wutticharoenwong V, Tempark T, Wananukul S (2018). Clinical features and treatment outcomes among children with Stevens-Johnson syndrome and toxic epidermal necrolysis: a 20-year study in a tertiary referral hospital. Dermatol Res Pract.

[11] Maity S, Banerjee I, Sinha R, Jha H, Ghosh P, Mustafi S (2020). Nikolsky's sign: a pathognomic boon. J Family Med Prim Care.

[12] Ross RK, Gerber JS, Willis ZI, Hersh AL, Kinlaw AC (2021). Outpatient fluoroquinolone use in children, 2000-2018. J Pediatric Infect Dis Soc.

[13] Vassallo C, Ruffo Di Calabria V, Isoletta E, Biscarini S, Di Filippo A, Brazzelli V (2021). Clinical and microbiological characteristics of reactive infectious mucocutaneous eruption: A case series of 5 patients. JAAD Case Rep.

